# Aetiology, Treatment and Outcomes of Pericarditis: Long-Term Data from a Longitudinal Retrospective Single-Centre Cohort

**DOI:** 10.3390/jcm13226900

**Published:** 2024-11-16

**Authors:** Andrea Silvio Giordani, Iris Bocaj, Cristina Vicenzetto, Anna Baritussio, Dario Gregori, Federico Scognamiglio, Honoria Ocagli, Renzo Marcolongo, Alida Linda Patrizia Caforio

**Affiliations:** 1 Unit of Cardiology, Department of Cardiac, Thoracic, Vascular Sciences and Public Health, University of Padova, 35122 Padova, Italy; 2 Unit of Biostatistics, Epidemiology and Public Health, Department of Cardiac, Thoracic, Vascular Sciences and Public Health, University of Padova, 35122 Padova, Italy

**Keywords:** acute pericarditis, anti-inflammatory therapy, interleukin-1 blockade, immunosuppressive therapy, cardioimmunology

## Abstract

**Background**. Pericarditis has a heterogeneous clinical spectrum and rate of relapse. Data on aetiology, real-life treatment strategies, and long-term course from contemporary pericarditis cohorts are lacking. **Methods**. Pericarditis patients referred to the Cardioimmunology Outpatient Clinic at Padua University Hospital in 2001–2020 were retrospectively included. Kaplan–Meier method was used for recurrence-free survival probability estimation. The appropriateness of treatment was assessed based on the European Society of Cardiology guidelines. **Results.** One-hundred forty-four patients (57% males, mean age 50 years) followed up for 18 months (IQR 7–45) were included; of those, 52% had acute, 35% recurrent, 8% incessant, and 5% chronic pericarditis; 9% had cardiac tamponade at diagnosis. Time to pericardial effusion resolution was 53 days (IQR 16–124); median medical treatment duration was 87 days (IQR 48–148). Treatment was readjusted following the ESC guidelines for nonsteroidal anti-inflammatory drugs in 29% of the cases, steroids in 12%, and colchicine in 25%. Eleven (8%) patients were treated with anti-IL1 agents. Recurrence-free survival probability was 86% at 1st-year follow-up, and 23 patients (16%) had at least one recurrence, with a mean of two relapses per patient. Compared to patients without recurrences, they had a higher frequency of cardiac tamponade (27% vs. 6%, *p* = 0.006) and left bundle branch block (14% vs. 1%, *p* = 0.034). Out of the 144 patients, 5 (3%) were diagnosed as having constrictive pericarditis at first evaluation at our clinic, underwent successful pericardiectomy, and are currently alive and asymptomatic. **Conclusions**. When treated following a guideline-based approach, pericarditis has a favourable evolution. A relevant quote of cases benefits from the treatment readjustment of previously prescribed medical therapy when not in line with ESC recommendations. Cases relapsing despite treatment readjustment should receive anti-IL1 therapies.

## 1. Introduction

Pericarditis, as an isolated/idiopathic disease and, less frequently, as a consequence of a pericardial injury or systemic immune-mediated disease (SID), is a common disease in clinical practice. According to the literature, acute pericarditis (AP) is the final diagnosis in about 4.5% of patients admitted to the emergency department because of chest pain [1], and pericardial effusion is found on fast point-of-care echocardiography in up to 14% of unselected patients accessing the emergency room because of dyspnoea [2].

Frequently, AP shows a rapid and complete response to nonsteroidal anti-inflammatory drugs (NSAIDs) [3]; however, in a relevant quote of patients, it tends to recur over prolonged periods, causing significant discomfort and the deterioration of life quality, especially in young patients [4,5,6]. AP may thus relapse one or several times, and the disease may convert from acute into recurrent (if the relapse happens after at least 4–6 weeks symptom-free interval), unceasing (if the acute phase lasts more than 4–6 weeks but less than 3 months) and chronic (>3 months of disease duration) [4]. The relapse rate may be successfully reduced by setting the appropriate therapeutic regimen from the first episode: if colchicine is not added to therapy from the first AP episode, the relapse rate may reach 38%, whilst its use can significantly reduce long-term hospital readmission for recurrences and the long-term recurrence risk. AP recurrences may occur in young, otherwise healthy patients, or in comorbid patients requiring a tailored etiological and therapeutic management, for instance, if pericarditis presents in association with a SID, is associated with specific infective agents (i.e., tuberculosis) or follows a pericardial injury (i.e., cardiac surgery) [7].

Since the early addition of colchicine to NSAIDs usually prevents relapses, progression to constrictive pericarditis (CP), a severe form with higher mortality even after pericardiectomy [8], is rare. Actually, AP relapses are usually attributed to a deranged individual immune reactivity [9,10]. Nevertheless, since many AP patients do not always receive appropriate therapy or manifest reduced compliance to treatment, it is plausible that, at least in some cases, rather than intrinsic reasons, recurrences could be the consequence of inadequate/inaccurate treatment. For instance, the early use of steroids has been found to be associated with subsequent disease recurrences and a longer course of recurrent pericarditis [11,12].

The 2015 European Society of Cardiology guidelines, as well as the previous 2004 version [13], establish a precise treatment protocol for acute and non-acute pericarditis [4]. For acute cases, a first-line combination therapy with NSAIDs and colchicine is indicated; the recommendations on drug choice, dosage, modality, and duration of tapering must be strictly followed, since recurrence may be related to excessively fast tapering [14]. In the modern management of AP, biological agents have been integrated into clinical practice: anti-IL1 agents, such as anakinra, have successfully changed the course of recurrent pericarditis, showing excellent profile of efficacy and safety, particularly in colchicine-resistant and steroid-dependent cases [15]. Nevertheless, long tapering is often needed, and recurrences may occur even after a long time after reaching a low dose of anakinra usage [16].

Yet, updated long-term data on the aetiology, type of treatment, and outcomes in contemporary cohorts of acute pericarditis are lacking. Thus, our aim was to define the frequency of different etiological types, the spectrum of clinical presentation, evolution, and prognosis, in terms of both death-free and relapse-free survival, of a large single-centre cohort of patients with AP. We also considered the spectrum, appropriateness, efficacy, and side effects of the treatment received by patients before and after referral to our centre and aimed to identify possible relapse predictors.

## 2. Materials and Methods

Complete clinical records of consecutive patients who were referred with a diagnosis of pericarditis to the Cardioimmunology Outpatient Clinic of the Padua University Hospital between May 2001 and May 2020 were retrospectively reviewed. Pericarditis course could be either acute, unceasing, recurrent, chronic, or constrictive; this classification was based upon the ESC recommendations [4]. Patients fulfilling the diagnostic criteria for clinically suspected myocarditis according to the ESC recommendations were excluded from this study [17]. Data regarding cardiological and immunological evaluation, electrocardiogram (ECG), Colour-Doppler transthoracic echocardiogram (CD-TTE), and medical or surgical treatment were considered. Data collection was carried out by Redcap^®^ (Research Electronic Data Capture) [18] according to a pre-established protocol that included diagnosis and follow-up data. In particular, the diagnosis section included detailed anamnesis (age, gender, ethnicity, family history of autoimmune diseases and cardiovascular diseases, personal history of autoimmune diseases, asthma, allergies, history of pericarditis, myocarditis and of acute viral infection in the six months before the diagnosis), clinical characteristics at presentation (symptoms, New York Heart Association (NYHA) class, serum C-reactive protein and troponin levels, electrocardiogram and CD-TTE features, and occurrence of cardiac tamponade) and information about treatment before referral to our hospital. The follow-up section included information about treatment after referral to our hospital, the occurrence of chest pain, NYHA class, levels of serum C-reactive protein (CRP), ECG changes, and CD-TTE and relapses. Pericarditis aetiology assessment was based on the ESC guidelines recommendations; with respect to infectious aetiology, in the absence of pericardial effusion analysis or specific microbiological findings, and in the presence of suggestive clinical findings, the cause of the first episode of acute pericarditis was presumed viral.

After referral to our centre, patients were treated according to the European Society of Cardiology (ESC) recommendations [4,13]: first-line therapy included the association of colchicine (0.5 mg once or bid according to body weight) and an NSAID (i.e., aspirin 750–1000 mg every 8 h or ibuprofen 600 mg every 8 h), which was slowly tapered only in the absence of symptoms and CRP abnormality, according to a pre-established protocol (i.e., Aspirin dosage was decreased of 200 mg/day every 2 weeks, and Ibuprofen of 200 mg/day every 2 weeks). Corticosteroids were introduced only in case of documented relapse slightly after, or during, NSAID tapering, or in case of contraindications to high-dose NSAIDs (i.e., severe kidney injury). Relapses were treated similarly to the first episode. From 2016, anti-IL1 agents were integrated in the treatment of recurrent, colchicine-resistant, and steroid-dependent cases; the initial dosage was 100 mg/day, tapered after 3–6 months by reducing the weekly dosage schedule by 1 dose per week.

Medical treatment for AP before referral to our centre was considered not in line with the ESC guidelines if at least one of the following criteria was met: (1) the dosage of the drug was too low in the absence of clear contraindications; (2) the combination of drugs was not in line with the 2015 ESC guidelines (i.e., colchicine was not used, or steroids were used as first-line therapy); and (3) tapering was too fast. In these cases, before introducing second- or third-line therapies, first-line drugs such as NSAIDs and colchicine were introduced or reintroduced following a guideline-based approach.

### Statistical Analysis

Continuous variables are reported as median (interquartile range (IQR)) or mean ± standard deviation (s.d.); categorical variables are reported as absolute numbers (percentage). Continuous variables were compared with Wilcoxon or Kruskal–Wallis tests, and categorical variables with chi-square and Fisher’s tests.

Time to pericardial effusion resolution was determined by tracking its presence or absence at each follow-up visit. The duration of an effusion episode was calculated from its first detection until the first visit where it was no longer present, providing in that way the cumulative duration of the effusion episode in days.

Treatment duration was calculated by tracking changes in medication combinations (e.g., NSAIDs, steroids, and colchicine) across follow-up visits. Each unique combination of medications defined a treatment period. The duration of each period was calculated from its start to end date, with the total duration assigned to the final visit of that period.

The distribution of the recurrence-free survival probability over time was estimated using the Kaplan–Meier method, while the impact of the covariates of interest on the outcome (relapse) was evaluated using Cox univariate analysis models. A *p*-value < 0.05 was considered statistically significant. Analyses were performed using the “rms” [19], “survival” [20], and “survminer” packages [21] in R software (version 4.3.3, available from https://www.R-project.org).

With regard to missing data, rather than applying specific imputation methods or exclusion criteria, we retained observations with missing values in their original form within the dataset to conduct the modelling estimations by employing listwise deletion, effectively conducting a complete case analysis for each analytical step.

This study was conducted according to the guidelines of the Declaration of Helsinki and approved by the local Ethics Committee (Comitato Etico per la Sperimentazione Clinica of Azienda Ospedale-Università di Padova, protocol number 002784, date of approval 2 April 2021).

## 3. Results

### 3.1. Patients’ Baseline Characteristics

The cohort consisted of 144 patients with pericarditis, 139 (97%) with AP and 5 (3%) with CP (Table 1). More than half of patients (57%) were male, and the median age at diagnosis was 50 years (IQR 34–64 years). All patients were Caucasian, except one African. Notably, 8 subjects had a family history of autoimmune diseases, and 12 had a concomitant autoimmune disease (3 with Hashimoto thyroiditis, 2 rheumatoid arthritis, 1 systemic lupus erythematosus, 1 psoriatic arthritis, 1 autoimmune diabetes, 1 undifferentiated connective tissue disease, and 1 ankylosing spondylitis). Immunological screening showed that 24 out of 55 patients tested positive for antinuclear antibodies (ANAs), while 5 out of 46 patients tested positive for extractable nuclear antigen (ENA) antibodies. Asthma was a comorbidity in 10 (7%) patients, and various allergies were reported in 34 (25%) patients.

Pericarditis aetiology was idiopathic/presumed viral in 112 subjects (78%), secondary to iatrogenic pericardial injury in 26 (18%), secondary to Dressler syndrome in 2 (1%), related to a SID in 3 (2%), and bacterial in 1 (1%). In 45 (39%) patients, clear viral infection symptoms were reported in the 6 months prior to pericarditis diagnosis. A relevant quote of the cohort (63 patients, 44%) had a remote or recent history of previous pericarditis episodes before referral to our centre. Overall, the clinical presentation was acute in 75 patients (52%), incessant in 11 (8%), recurrent in 51 (35%) and chronic in 8 (5%). At diagnosis, symptoms of heart failure were rarely reported: NYHA class at diagnosis was III in seven (5%) and IV in six (5%) subjects. Cardiac tamponade occurred in 13 (9%) patients. Serum CRP levels at diagnosis were abnormal in 101 patients (91%), with a median value of 12.3 mg/dL (IQR, 6.9–19.0 mg/dL, reference value < 5 mg/dL), and 21 patients (24%) had abnormal troponin levels.

**Figure 1:**
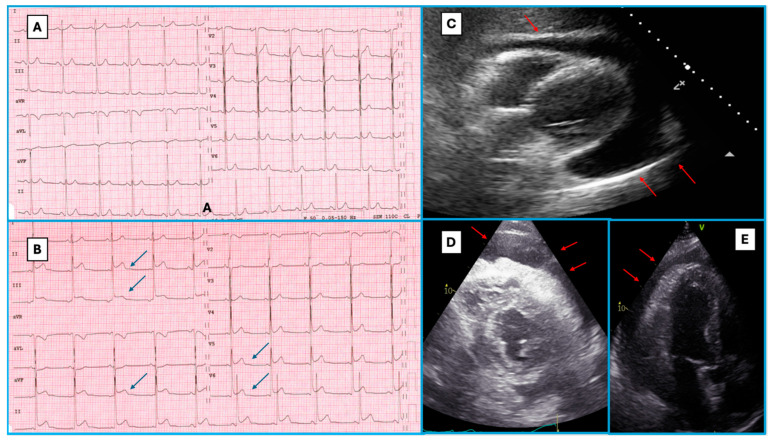
ECG and echocardiographic findings in different patients with acute pericarditis. Typical ECG findings in a patient with acute pericarditis, presenting to the emergency department because of continuous chest pain worsening when breathing and easing when leaning forwards. Panel (**A**) shows a previous normal ECG of the same patient, and Panel (**B**) shows the ECG registered at the admission for acute pericarditis, with concave ST-segment elevation in the inferolateral leads (blue arrows) with associated PR-segment depression. Panels (**C**) (subcostal view), (**D**) (parasternal short axis view), and (**E**) (apical 3 chamber view) show the echocardiographic features of severe pericardial effusion (anechoic space between the pericardial layers, red arrows).

Concerning ECG at diagnosis, atrial fibrillation was the cardiac rhythm at diagnosis in 11 subjects. ECG findings suggestive of pericarditis were observed in a sizable quote of cases: concave, diffuse ST-segment elevation without reciprocal ST-segment depression was observed in 60 (62%) cases. Moreover, nine patients (9%) showed a typical PR-segment depression. Other repolarisation abnormalities (T-wave inversion) were observed in a minority of cases (six patients had inverted T-waves in the anterior leads, four in the inferior ones, and four in the lateral ones). Bundle branch block (BBB) was present in three patients (3.1%). Regarding CD-TTE findings, LV function was overall preserved, and pericardial effusion was noticed in 97 (67%) cases: mild in 51 (44%) patients, moderate in 26 (23%), and severe in 20 (17%) (Figure 1). Among the latter group, 17 patients required a pericardiocentesis. Cardiac magnetic resonance (CMR) was performed in a minority of cases (16/144), frequently showing pericardial effusion (44%), pericardial oedema (50%), and/or pericardial late gadolinium enhancement (LGE, 50%).

### 3.2. Follow-Up Data

**Figure 2:**
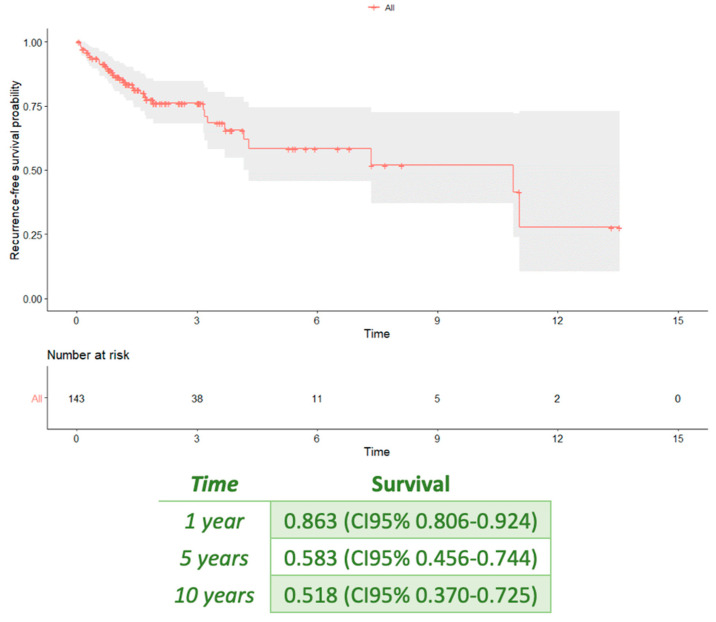
Recurrence-free survival probability.

The median follow-up duration was 18 months (IQR, 7–45 months) (Figure 2). No patients died during follow-up; one patient was lost to follow-up.

The median time for pericardial effusion resolution was 53 days (IQR 16–124). Twenty-three (16%) patients had at least one pericarditis recurrence, and the mean number of relapses per patient was two (Table 2). The estimated recurrence-free survival probability was 0.863 (95% CI, 0.806–0.924) in the 1st year, 0.583 (95% CI, 0.456–0.744) in the 5th year, and 0.518 (95% CI, 0.370–0.725) in the 10th year.

At the last follow-up, only 5 patients (3%) reported NYHA class II, only 7 (5%) patients complained of pericarditic chest pain, and 10 (9%) had abnormal CRP serum level (median value 0.79 mg/dL, IQR 0.60–1.94 mg/dL). At the last follow-up, the ECG was normalised in almost all cases; in only three cases (2%), inverted T-waves were present in the anterior leads: one (1%) in the lateral ones and two (2%) in the inferior ones. Pericardial effusion was absent in 92% of cases; when present, it was mainly mild (10 cases) and severe in only 1 case.

At the last follow-up, 107 out of 111 patients (96%) with acute/recurrent pericarditis obtained complete remission following standard treatment. Only four patients (3%) experienced a relapse in the previous 3 months and three (2%) reported being intolerant to one or more drugs. In total, 11 patients (8%) entered steady remission only after switching to interleukin-1 (IL-1) blocking therapy.

### 3.3. Characteristics of Medical Treatment for AP

Following referral to our centre, therapy was modified or readjusted according to ESC guidelines in 68% of the cases. Medical treatment had a duration of 87 days (IQR 48–148). At the first episode of pericarditis, before referral to our outpatient clinic, 92 patients (64%) received ibuprofen, 18 (13%) indomethacin, 10 (7%) aspirin, and 2 (1%) other NSAIDs; colchicine was administered to 78 (54%) patients and corticosteroids to 27 (19%). Initial treatment with NSAIDs was not in line with ESC 2015 guidelines for 43 (31%) patients, while the introduction of steroids appeared inappropriate in 17 (12%), and colchicine was not prescribed in 39 (28%). Before referral to our outpatient clinic, 18 patients (12%) reported drug-related collateral effects: 7 patients to colchicine (diarrhoea), 9 to NSAIDs (gastric/abdominal pain, haematological abnormalities, hepatic toxicity, and allergic reactions), and 2 to corticosteroids. Three patients underwent surgery for a pleuropericardial window. Eleven (8%) patients, after the failure of first- and/or second-line guideline-based treatment, were treated with anti-IL1 agents, with stable and durable control of the disease.

### 3.4. Constrictive Pericarditis Cases

**Figure 3:**
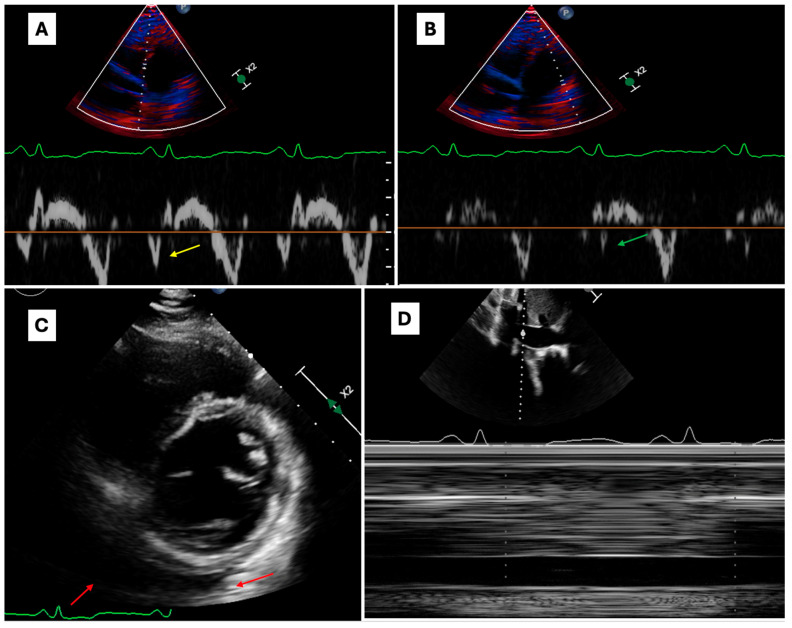
Echocardiographic findings of a patient with constrictive pericarditis. Echocardiographic findings of a patient with long-standing worsening right-sided heart failure. Panels (**A**,**B**) show an “annulus reversus” pattern on tissue Doppler imaging (TDI) evaluation (medial e’ velocity, yellow arrow; > lateral e’ velocity, green arrow; on same velocity reference scale). Panel (**C**) shows moderate circumferential pericardial effusion, more represented at the left ventricular posterior wall (red arrows). Panel (**D**) shows dilated and hypo-collapsible inferior vena cava.

Five Caucasian patients (four male, median age at diagnosis 52 years, IQR 31–60 years) had CP (Figure 3). In two cases, patients reported an acute viral infection in the six months before diagnosis. One patient had a history of recurrent pericarditis and another had a history of clinically suspected myocarditis, while no one had concomitant immune-mediated diseases, allergies, or asthma. The aetiology was identified as idiopathic/presumed viral in three subjects and iatrogenic in two. Serum CRP was determined in four subjects and resulted in abnormalities in three (mean value 11.1 mg/dL, IQR, 2.4–22.9 mg/dL). Two patients underwent cardiac catheterisation showing a typical “dip–plateau” ventricular filling pattern. At the onset, two patients were treated with indomethacin and colchicine; one patient with ibuprofen, indomethacin, colchicine, and corticosteroids; one received only ibuprofen; and one patient was initially treated with indomethacin and then received corticosteroids. All patients underwent pericardiectomy and survived. In two cases, the results of the histological examination of the pericardium were available, showing the fibrotic thickening of the pericardium, and no findings were suggestive of infectious aetiology in either case.

### 3.5. Differences Between Patients With and Without Pericarditis Relapses

During follow-up, 23 patients (16%) experienced at least one pericarditis relapse. With respect to patients without recurrences, patients with recurrences did not show significant differences in terms of sex, age, type of initial treatment for pericarditis, or biochemical markers (Table 3). Notably, the only two significant differences were pericardial tamponade at diagnosis (27% in patients with recurrences vs. 6% in patients without recurrences, *p* = 0.006) and LBBB (14% in patients with recurrences vs. 1% in patients without recurrences, *p* = 0.034). Nevertheless, by univariate analysis, no significant correlation was observed between clinical covariates and the risk of recurrence (Supplementary Table S1).

## 4. Discussion

Our data confirm that pericarditis is a benign disease, often of idiopathic origin but can present a relevant rate of relapse (16%, among our patients). In our cohort, severe onset with tamponade at diagnosis was associated with recurrences, as well as the presence of LBBB at baseline ECG. In addition, a first-line treatment that was not completely adherent to international recommendations was often encountered in our real-world scenario (68% of our cohort of patients referred to a specialised outpatient clinic), reflecting the need for the diffusion of updated recommendations among medical specialists that prescribe first-line treatment to patients with a first pericarditis episode.

Following adherence to standard treatment ESC guidelines, 96% of our patients with acute/recurrent pericarditis obtained complete remission, while only 11 patients required IL-1 blocking therapy. All patients with CP underwent uncomplicated pericardiectomy. This confirms that, when appropriately treated and following careful and personalised follow-up, pericarditis has usually a favourable evolution, showing a good prognosis even in its most adverse or uncommon presentations, such as recurrent AP and CP.

In keeping with a previous case–control study on infections in AP, an acute respiratory or gastrointestinal viral infection preceded the clinical onset of pericarditis in 39% of our patients [22]. Similarly to other organ-specific immune-mediated diseases, initial pericardial inflammation could be triggered by a viral infection, while relapses are likely sustained by a deranged/redundant individual immune response [9,10,23,24]. Among our patients, a concomitant immune-mediated disease was detected in 12 subjects (9%), while 29 subjects revealed circulating autoantibodies, suggesting the possible influence of an autoimmune background on both the appearance and recurrence of pericardial inflammation. Conversely, allergies and asthma were not associated with a higher risk of relapse, probably because, unlike pericardial inflammation, which is predominantly driven by IL-1, a T-helper-1 cytokine, allergic diseases are predominantly T-helper-2-related conditions [25]. In line with this assumption, following anti-IL-1 receptor agent blockade, we observed prompt and stable clinical remission in all the 11 patients treated with anakinra, with multi-refractory recurrent AP.

Also, the incidence of iatrogenic and bacterial pericarditis observed in our patients is in line with medical literature, i.e., between 17% and 20% and <1% [26,27], respectively, while we did not observe any tubercular form, probably due to a decline in tubercular infection in our region. No patients with a malignant pericardial effusion were referred to our centre in the considered time interval, perhaps because, as a tertiary referral centre, we predominantly see patients with a confirmed diagnosis of long-lasting and/or refractory pericarditis. The same bias might also account for the increased incidence (43%) of recurrent and incessant forms observed in our patients, as well as the high proportion of cases with post-pericardial injury, essentially post-cardiac surgery pericarditis cases (26 patients, 18%).

The serum level of CRP was elevated in 91% of our patients at diagnosis, confirming to be a reliable and sensitive diagnostic marker, as well as a useful clinical parameter to adjust treatment during patients’ follow-up. The troponin level at diagnosis was abnormal in 21 subjects (24%). This finding is in line with the literature [24], but when particularly or persistently elevated, it requires a differential diagnosis with coronary artery disease and myocarditis. Actually, both pericarditis and myocarditis can start with sudden onset precordial pain and concomitant CRP and troponin elevations, along with overlapping ECG changes [4,28]; additionally, both pericarditis and myocarditis can be caused by a cardiotropic virus [28]. In our cohort, ECG showed typical signs of pericarditis in more than 60% of cases; this is in line with previously reported data [29]. Remarkably, in our cohort a quote of patients showed T-wave inversion on baseline ECG (in six cases, T-wave inversion was observed in the anterior ECG leads; in four cases, in the inferior leads; and in 4 cases, in the lateral leads) in the absence of other findings suggestive of clinically suspected myocarditis; in fact, it is already established that T-wave inversion may develop after ST-segment elevation in acute pericarditis, even in the absence of epicardial involvement, with relevant implications for correct diagnosis and treatment [30].

In addition, in keeping with the literature [31,32], cardiac tamponade at clinical onset was more frequent in patients with subsequent recurrences than in patients without recurrences (27% vs. 6%, *p* = 0.006). This may reflect a more aggressive disease with rapid pericardial effusion formation at onset; nevertheless, in the absence of haemodynamic instability, performing the pericardiocentesis of a stable pericardial effusion has shown no benefit in terms of specific diagnosis or treatment strategies in previous cohorts [33].

Regarding the medical treatment that patients were taking before referral to our centre, a relevant quote of patients had not received medical treatment in line with the latest ESC guidelines: notably, 43 patients (31%) were receiving inappropriate NSAID therapy in terms of daily dosage and/or duration; 17 patients (12%) inappropriately received corticosteroids; and, despite the absence of known contraindications, colchicine therapy was omitted in 39 (28%). This may be due to the fact that the prompt clinical response observed in the vast majority of AP patients following the initiation of anti-inflammatory therapy, no matter if with NSAIDs and/or corticosteroids, can give the misleading impression of an early and complete resolution of pericarditis, encouraging the premature tapering and discontinuation of therapy. Furthermore, frequently, AP patients do not undergo adequate follow-up, nor do they receive adequate information/education about treatment and disease self-management. With this purpose, when entering follow-up at our centre, on top of standard pharmacological therapy, all patients receive careful education about their disease and treatment and are actively involved in their clinical risk management in order to reinforce motivation and adherence to treatment to reduce its side effects and prevent therapeutic and behavioural mistakes able to promote relapses.

It is conceivable that, to some extent, treatment inaccuracies may contribute to feeding the vicious circle of relapses, transforming a usually transient and otherwise nonthreatening clinical syndrome into a chronic, recurrent, and invalidating disease. Conversely, when treated according to ESC guidelines, in our experience, AP usually resolved in the vast majority of patients and never behaved as a life-threatening disease. As a matter of fact, in about 90% of our AP patients, the simple resumption and/or adequation of anti-inflammatory therapy with NSAIDs and colchicine in line with the ESC guidelines proved effective in reaching a steady resolution of pericardial inflammation. Therefore, only 11 (8%) of our AP patients showed a true treatment-refractory form that required a second-line therapy. The introduction of an immunosuppressive agent or, in line with the more recent evidence, anti-IL-1 blockade with anakinra, produced an effective clinical response. In particular, 8 out of 11 patients (73%) who received immunosuppressive therapy obtained complete disease resolution, while 3 (27%) entered stable clinical remission only following therapy with anakinra. Before the introduction of IL-1R blocking therapy, treatment with azathioprine and or other immunosuppressive/immunomodulating agents, such as cyclosporine A, methotrexate, and high-dose intravenous immunoglobulins (HDIVIGs), showed a certain efficacy in patients with prolonged, cortico-dependent, recurrent AP [34,35,36]. Furthermore, immunosuppressive therapy seems more appropriate in the treatment of pericarditis secondary to SIDs. During the study period, 11 patients (8%) with AP refractory to standard treatment were treated with anakinra, obtaining complete clinical remission and discontinuation of previous therapy. Yet, despite progressive, individualised drug tapering, so far, none of the patients receiving anakinra was able to achieve complete discontinuation of therapy [25].

The observed incidence of side effects was 6% for NSAIDs, 3% for colchicine, and 1% for corticosteroids, with the most frequently reported being epigastric pain. The use of azathioprine was associated with pneumonia in one case, while patients who started anakinra reported initial, transient urticarial manifestations at the injection site. However, the correct implementation of ESC treatment guidelines can be difficult in patients who cannot receive standard NSAIDs and colchicine therapy due to severe intolerance, concomitant drug interaction, or comorbidity (e.g., critical liver and/or renal dysfunction, coagulopathy, gastric inflammation/ulceration, chronic bowel inflammatory diseases, etc.). In these patients, the careful introduction of a low-dose corticosteroid therapy, although usually contraindicated in AP, may still represent a safe alternative.

Patients with CP represented only 3% of our cohort, probably because this elusive disease is rarely preceded by an acute exudative form (only one case in our cohort) but also because these patients often follow dedicated diagnostic and therapeutic tracks. It is conceivable that compared to AP, the prevalent fibrotic evolution of CP is likely related to the production of fibrogenic TH2-derived cytokines, while no patient had a serum IgG4 elevation or signs and symptoms related to IgG4 disease [37,38]. All our CP patients underwent uncomplicated pericardiectomy followed by a satisfactory clinical recovery, suggesting that an early diagnosis and follow-up may have relevant prognostic implications in CP as well.

## 5. Study Limitations

This study has potential limitations. First, our observational retrospective study was conducted in a tertiary hub centre for pericarditis, potentially affecting patients’ characteristics, and therefore they may not be representative of unselected populations; nevertheless, this is a real-life population. Secondly, the reduced number of relapses observed in our study population through the follow-up period accounts for the low statistical power of risk stratification. Thirdly, since all patients received the initial diagnosis of pericarditis at other centres, a complete collection of clinical data was not possible for all cases. Finally, considering the relatively limited sample size and the presence of missing data, future research might benefit from implementing different statistical approaches.

## 6. Conclusions

In conclusion, when treated according to ESC guidelines and with an accurate and personalised follow-up, pericarditis has a good prognosis in the majority of patients, even in case of previous recurrences. A relevant quote of cases may not have been treated in line with ESC recommendations from the first episode and benefitted from treatment readjustment. Cases relapsing despite treatment readjustment should receive anti-IL1 agents to achieve stable remission.

## Figures and Tables

**Table 1:** Patients’ baseline characteristics.

Characteristics	Total Population (*n* = 144)	Missing Data (N)
Ethnicity		0
African, *n* (%)	1 (1%)	
Caucasian, *n* (%)	143 (99%)	
Age at diagnosis (years), IQR/mean ± s.d.	34–50–64/49 ± 20	0
Gender		0
Female, *n* (%)	62 (43%)	
Male, *n* (%)	82 (57%)	
Family history		
Immune-mediated diseases, *n* (%)	8 (20%)	103
Cardiovascular diseases, *n* (%)	30 (52%)	86
Personal history		20
Acute viral infection (6 months pre-diagnosis), *n* (%)	45 (39%)	
Autoimmune diseases, *n* (%)	12 (9%)	
Allergies, *n* (%)	34 (25%)	
Asthma, *n* (%)	10 (7%)	
Pericarditis history, *n* (%)	63 (44%)	0
Myocarditis history, *n* (%)	3 (2%)	0
Clinical presentation		0
Acute, *n* (%)	75 (52%)	
Incessant, *n* (%)	11 (8%)	
Recurrent, *n* (%)	51 (35%)	
Chronic, *n* (%)	7 (5%)	
Aetiology		0
Bacterial, *n* (%)	1 (1%)	
Dressler, *n* (%)	2 (1%)	
Iatrogenic pericardial injury, *n* (%)	26 (18%)	
Idiopathic/presumed viral, *n* (%)	112 (78%)	
SIDs (systemic immune-mediated diseases), *n* (%)	3 (2%)	
Cardiac tamponade, *n* (%)	13 (9%)	3
Symptoms before diagnosis, *n* (%)	126 (95%)	11
Palpitations, *n* (%)	20 (16%)	19
Syncope, *n* (%)	10 (8%)	20
Chest pain, *n* (%)	114 (92%)	10
NYHA class		14
I, *n* (%)	88 (68%)	
II, *n* (%)	29 (22%)	
III, *n* (%)	7 (5%)	
IV, *n* (%)	6 (5%)	
Pericardial biopsy, *n* (%)	2 (2%)	0
ECG data		21
Sinus rhythm, *n* (%)	86 (88%)	46
Atrial fibrillation, *n* (%)	11 (11%)	0
Other (pacemaker-induced rhythm), *n* (%)	1 (1%)	0
Concave ST elevation, *n* (%)	60 (62%)	47
Anterior inverted T-waves, *n* (%)	6 (6%)	47
Lateral inverted T-waves, *n* (%)	4 (4%)	47
Inferior inverted T-waves, *n* (%)	4 (4%)	47
PR depression, *n* (%)	9 (9%)	47
Echocardiography Findings		44
LA (mm), IQR/mean ± s.d.	30.8–34.0–40.2/34.8 ± 9.6	
LA volume (mL/m^2^), IQR/mean ± s.d.	25.0–28.0–36.0/30.7 ± 9.3	
LVEDV (mL/m^2^), IQR/mean ± s.d.	45–53–63/54 ± 14	
LVEF (%), IQR/mean ± s.d.	56.0–60.0–64.5/59.7 ± 7.1	
RVEDA (cm^2^), IQR/mean ± s.d.	15.9–19.3–22.0/19.3 ± 4.9	
FAC (%), IQR/mean ± s.d.	42.5–47.0–50.0/46.5 ± 6.8	
CMR findings		128
Pericardial effusion, *n* (%)	7/16 (44%)	
Pericardial oedema, *n* (%)	8/16 (50%)	
Pericardial LGE	8/16 (50%)	
Pericardial Effusion		0
Absent, *n* (%)	18 (16%)	
Mild, *n* (%)	51 (44%)	
Moderate, *n* (%)	26 (23%)	
Severe, *n* (%)	20 (17%)	
Pericarditis treatment		0
Ibuprofen, *n* (%)	92 (64%)	
Colchicine, *n* (%)	78 (54%)	
Indomethacin, *n* (%)	18 (13%)	
ASA, *n* (%)	10 (7%)	
NSAIDs, *n* (%)	2 (1.4%)	
Steroids, *n* (%)	27 (19%)	
Abnormal CRP *n* (%)	101 (91%)	1
CRP levels mg/dL, IQR/mean ± s.d.	6.9–12.3–19.0/19 ± 38	
Abnormal troponin, *n* (%)	21 (24%)	10
Autoantibodies		115
ANAs (antinuclear antibodies), positive, *n* (%)	24 (44%)	
ENA antibodies, positive, *n* (%)	5 (11%)	

CMR: cardiac magnetic resonance; LGE: late gadolinium enhancement; SIDs: systemic immune-mediated diseases; NYHA: New York Heart Association; LA: left atrium; LVEDV: left ventricular end-diastolic volume; LVEF: left ventricular ejection fraction; RVEDA: right ventricular end-diastolic area; FAC: fractional area change; AHA: anti-heart antibodies; ANA: antinuclear antibodies.

**Table 2:** Last follow-up status.

Last Follow-Up	Total Population *n* = 143	Missing Data, *n*
Follow-up time (months), IQR/mean ± s.d.	18 (7–45)	1
Patients with at least one relapse during follow-up *n* (%)	23 (16%)	0
Number of relapses per patient, mean ± s.d.	2 (2)	0
NYHA class:	138 (97%)	11
I, *n* (%)	5 (3%)	
II, *n* (%)	7 (5%)	
Chest pain, *n* (%)	10 (9%)	0
Abnormal CRP, *n* (%)		0
Ongoing treatment:		0
Ibuprofen, *n* (%)	6 (4%)	
Indomethacin, *n* (%)	4 (3%)	
Aspirin, *n* (%)	1 (1%)	
Other NSAIDs, *n* (%)	2 (1%)	
Colchicine, *n* (%)	38 (27%)	
Corticosteroids, *n* (%)	9 (6%)	
Anakinra, *n* (%)	11 (8%)	
Hydroxychloroquine, *n* (%)	5 (3%)	
Immunosuppressive drugs, *n* (%)	3 (2%)	
IVIG, *n* (%)	0 (0%)	
Ongoing treatment response, *n* (%)	107 (96%)	
Time for pericardial effusion resolution (days), median (IQR)	53 (16–124)	
Duration of treatment, (days), median (IQR)	87 (48–148)	
ECG data:		1
Sinus rhythm, *n* (%)	120 (98%)	
Anterior inverted T-waves, *n* (%)	3 (2%)	
Lateral inverted T-waves, *n* (%)	1 (1%)	
Inferior inverted T-waves, *n* (%)	2 (2%)	
Echo data:		1
LA (mm), IQR/mean ± s.d.	31.0–36.0–41.0/35.9 ± 7.5	
LA volume (mL/m^2^), IQR/mean ± s.d.	14–21–27/23 ± 10	
LVEF (%), IQR/mean ± s.d.	62–67–71/67 ± 6	
RVEDA (cm^2^), IQR/mean ± s.d.	15–17–20/17 ± 4	
FAC (%), IQR/mean ± s.d.	47.0–50.0–55.0/50.6 ± 7.5	
Pericardial effusion:		
Absent, *n* (%)	119 (92%)	
Mild, *n* (%)	10 (8%)	
Severe, *n* (%)	1 (1%)	

NYHA: New York Heart Association; CRP: C-reactive protein; NSAIDs: nonsteroidal anti-inflammatory drugs; IVIG: intravenous immunoglobulins; LA: left atrium; LVEF: left ventricular ejection fraction; RVEDA: right ventricular end-diastolic area; FAC: fractional area change.

**Table 3:** Differences between patients with and without relapses.

Characteristics	No Relapse (*n* = 121)	Yes Relapse (*n* = 23)	*p*-Value
Ethnicity			>0.9
African, *n* (%)	1 (0.8%)	0 (0%)	
Caucasian, *n* (%)	120 (99%)	23 (100%)	
Gender			0.3
Female, *n* (%)	50 (41%)	12 (52%)	
Male, *n* (%)	71 (59%)	11 (48%)	
Age (years), mean (±s.d.)	55 (19)	51 (16)	0.3
Allergy, *n* (%)	31 (27%)	3 (14%)	0.2
Asthma, *n* (%)	8 (6.9%)	2 (9.5%)	0.7
Pericarditis history			0.044
No, *n* (%)	72 (60%)	8 (36%)	
Yes, *n* (%)	49 (40%)	14 (64%)	
Myocarditis history, *n* (%)	1 (0.8%)	2 (8.7%)	0.068
Aetiology			0.7
Bacterial, *n* (%)	1 (0.8%)	0 (0%)	
Dressler, *n* (%)	2 (1.7%)	0 (0%)	
Iatrogenic pericardial injury, *n* (%)	23 (19%)	3 (13%)	
Idiopathic/presumed viral, *n* (%)	93 (77%)	19 (83%)	
SIDs, *n* (%)	2 (1.7%)	1 (4.3%)	
Clinical presentation			
Acute, *n* (%)	65 (54%)	10 (43%)	0.4
Unceasing, *n* (%)	8 (6.6%)	3 (13%)	0.4
Recurrent, *n* (%)	41 (34%)	10 (43%)	0.4
Chronic, *n* (%)	8 (6.6%)	0 (0%)	0.4
Inappropriate treatment with NSAIDs, *n* (%)	34 (29%)	9 (43%)	0.2
Inappropriate treatment with steroids, *n* (%)	14 (12%)	3 (15%)	0.7
Appropriate treatment with colchicine, *n* (%)	89 (75%)	11 (55%)	0.068
Tamponade at diagnosis	7 (6%)	6 (27%)	0.006
NYHA class			0.4
I, *n* (%)	77 (69%)	11 (58%)	
II, *n* (%)	24 (22%)	5 (26%)	
III, *n* (%)	5 (4.5%)	2 (11%)	
IV, *n* (%)	5 (4.5%)	1 (5.3%)	
Palpitations, *n* (%)	18 (17%)	2 (11%)	0.7
Syncope, *n* (%)	9 (8.6%)	1 (5.3%)	>0.9
Chest pain, *n* (%)	95 (90%)	19 (100%)	0.4
Left-sided heart failure, *n* (%)	7 (6.3%)	2 (10%)	0.6
Right-sided heart failure, *n* (%)	6 (5.4%)	1 (5.3%)	>0.9
Troponin levels			0.3
Abnormal, *n* (%)	17 (22%)	4 (36%)	
Normal, *n* (%)	61 (78%)	7 (64%)	
C-reactive protein (CRP)			0.066
Abnormal, *n* (%)	86 (93%)	15 (79%)	
Normal, *n* (%)	6 (6.5%)	4 (21%)	
Pericardiocentesis at diagnosis, *n* (%)	13 (12%)	4 (21%)	0.3
Pericardial biopsy, *n* (%)	2 (1.9%)	0 (0%)	>0.9
Pericardial window procedure, *n* (%)	1 (0.9%)	2 (11%)	0.059

## Data Availability

The raw data supporting the conclusions of this article will be made available by the authors on request.

## Supplementary Materials

The following supporting information can be downloaded at: https://www.mdpi.com/article/10.3390/jcm13226900/s1, Table S1. Univariate regression analysis for pericarditis relapse. Table S2. STROBE Statement—checklist of items that should be included in reports of observational studies.
