# Supplementary material for: Aetiology, Treatment and Outcomes of Pericarditis: Long-Term Data from a Longitudinal Retrospective Single-Centre Cohort
Source: J Clin Med. 2024 Nov 16;13(22):6900. doi: 10.3390/jcm13226900 (PMC11595198; doi:10.3390/jcm13226900)
Supplement: Supplementary file 1 [file jcm-13-06900-s001.zip › jcm-3292230-Table S1.pdf]

Supplementary Table S1 – Univariate regression analysis for pericarditis relapse.

Variable	**Comparison**	**Hazard Ratio**	**p-value**
Immune mediated disease	Yes:No	1.91	0.1923
Gender	Female:Male	0.7386	0.3786
Age at diagnosis	---	0.6889	0.1682
Allergy	Yes:No	0.5207	0.1486
Asthma	Yes:No	0.5584	0.428
Aetiology	Other:Idiop/presumed viral	1.383	0.4056
Clinical presentation	Other:Acute	0.9905	0.9794
Tamponade at diagnosis	Yes:No	1.977	0.1341
Pericarditis history	Yes:No	1.335	0.4413
NYHA at diagnosis	III-IV:I+II	1.118	0.8551
Inappropriate NSAID treatment	Yes:No	1.222	0.5769
Inappropriate steroids treatment	Yes:No	0.4521	0.1497
Appropriate colchicine treatment	No:Yes	1.296	0.4743
Troponin	Abnormal:Normal	1.874	0.2194
Left sided heart failure	Yes:No	1.346	0.6863
Right sided heart failure	Yes:No	0.679	0.7044
Pericardial effusion	Absent:Present	0.7264	0.6093
Anti-nuclear antibodies	Yes:No	0.7519	0.6447
