# Supplementary material for: Aetiology, Treatment and Outcomes of Pericarditis: Long-Term Data from a Longitudinal Retrospective Single-Centre Cohort
Source: J Clin Med. 2024 Nov 16;13(22):6900. doi: 10.3390/jcm13226900 (PMC11595198; doi:10.3390/jcm13226900)
Supplement: Supplementary file 1 [file jcm-13-06900-s001.zip › jcm-3292230-Table S2.pdf]

Table S2. STROBE Statement—checklist of items that should be included in reports of observational studies

	**Item No.**	**Recommendation**	**Page No.**	**Relevant text from manuscript**
**Title and abstract**	1	(*a*) Indicate the study's design with a commonly used term in the title or the abstract	1	Line 24
		(*b*) Provide in the abstract an informative and balanced summary of what was done and what was found	1	Lines 25-39
**Introduction**				
Background/rationale	2	Explain the scientific background and rationale for the investigation being reported	3-4	Lines 49-85
Objectives	3	State specific objectives, including any prespecified hypotheses	5	Lines 87-91
**Methods**				
Study design	4	Present key elements of study design early in the paper	5	Lines 93-95
Setting	5	Describe the setting, locations, and relevant dates, including periods of recruitment, exposure, follow-up, and data collection	5-6	Lines 95-121
Participants	6	(*a*) *Cohort study*—Give the eligibility criteria, and the sources and methods of selection of participants. Describe methods of follow-up<br>*Case-control study*—Give the eligibility criteria, and the sources and methods of case ascertainment and control selection. Give the rationale for the choice of cases and controls<br>*Cross-sectional study*—Give the eligibility criteria, and the sources and methods of selection of participants	5	Lines 93-95
		(*b*) *Cohort study*—For matched studies, give matching criteria and number of exposed and unexposed<br>*Case-control study*—For matched studies, give matching criteria and the number of controls per case	N.A.	N.A.
Variables	7	Clearly define all outcomes, exposures, predictors, potential confounders, and effect modifiers. Give diagnostic criteria, if applicable	5-6	Lines 93-120
Data sources/ measurement	8*	For each variable of interest, give sources of data and details of methods of assessment (measurement). Describe comparability of assessment methods if there is more than one group	7	Lines 126-131
Bias	9	Describe any efforts to address potential sources of bias	7	Lines 137-145
Study size	10	Explain how the study size was arrived at	N.A.	N.A.

Continued on next page

Quantitative variables	11	Explain how quantitative variables were handled in the analyses. If applicable, describe which groupings were chosen and why	7	Line 133
Statistical methods	12	(*a*) Describe all statistical methods, including those used to control for confounding	7-8	Lines 133-153
		(*b*) Describe any methods used to examine subgroups and interactions	8	Lines 141-147
		(*c*) Explain how missing data were addressed	8	Line 151-153
		(*d*) *Cohort study*—If applicable, explain how loss to follow-up was addressed<br>*Case-control study*—If applicable, explain how matching of cases and controls was addressed<br>*Cross-sectional study*—If applicable, describe analytical methods taking account of sampling strategy	N.A.	N.A.
		(*e*) Describe any sensitivity analyses	N.A.	N.A.
**Results**				
Participants	13*	(a) Report numbers of individuals at each stage of study—eg numbers potentially eligible, examined for eligibility, confirmed eligible, included in the study, completing follow-up, and analysed		
		(b) Give reasons for non-participation at each stage	N.A.	N.A.
		(c) Consider use of a flow diagram	N.A.	N.A.
Descriptive data	14*	(a) Give characteristics of study participants (eg demographic, clinical, social) and information on exposures and potential confounders	8	Line 159
		(b) Indicate number of participants with missing data for each variable of interest	24, 27	Table 1 and 2: missing data are specified for each variable
		(c) *Cohort study*—Summarise follow-up time (eg, average and total amount)	9	Line 192
Outcome data	15*	*Cohort study*—Report numbers of outcome events or summary measures over time	9-10	Lines 192-207
		Case-control study—Report numbers in each exposure category, or summary measures of exposure	N.A.	N.A.
		Cross-sectional study—Report numbers of outcome events or summary measures	N.A.	N.A.
Main results	16	(*a*) Give unadjusted estimates and, if applicable, confounder-adjusted estimates and their precision (eg, 95% confidence interval). Make clear which confounders were adjusted for and why they were included	N.A.	N.A.
		(*b*) Report category boundaries when continuous variables were categorized	N.A.	N.A.
		(*c*) If relevant, consider translating estimates of relative risk into absolute risk for a meaningful time period	N.A.	N.A.

Continued on next page

Other analyses	17	Report other analyses done—eg analyses of subgroups and interactions, and sensitivity analyses	N.A.	N.A.
**Discussion**				
Key results	18	Summarise key results with reference to study objectives	11-12	Lines 245-251
Limitations	19	Discuss limitations of the study, taking into account sources of potential bias or imprecision. Discuss both direction and magnitude of any potential bias	16	Lines 356-364
Interpretation	20	Give a cautious overall interpretation of results considering objectives, limitations, multiplicity of analyses, results from similar studies, and other relevant evidence	12-13-14	Lines 252-322
Generalisability	21	Discuss the generalisability (external validity) of the study results	16	Lines 356-360
**Other information**				
Funding	22	Give the source of funding and the role of the funders for the present study and, if applicable, for the original study on which the present article is based	17	Line 378

*Give information separately for cases and controls in case-control studies and, if applicable, for exposed and unexposed groups in cohort and cross-sectional studies.

Note: An Explanation and Elaboration article discusses each checklist item and gives methodological background and published examples of transparent reporting. The STROBE checklist is best used in conjunction with this article (freely available on the Web sites of PLoS Medicine at http://www.plosmedicine.org/, Annals of Internal Medicine at http://www.annals.org/, and Epidemiology at http://www.epidem.com/). Information on the STROBE Initiative is available at www.strobe-statement.org.
